# The Current and Future Therapies of Bone Regeneration to Repair Bone Defects

**DOI:** 10.1155/2012/148261

**Published:** 2012-03-13

**Authors:** Eijiro Jimi, Shizu Hirata, Kenji Osawa, Masamichi Terashita, Chiaki Kitamura, Hidefumi Fukushima

**Affiliations:** ^1^Division of Molecular Signaling and Biochemistry, Department of Biosciences, Kyushu Dental College, 2-6-1 Manazuru, Kokurakita-ku, Kitakyushu, Fukuoka 803-8580, Japan; ^2^Center for Oral Biological Research, Kyushu Dental College, 2-6-1 Manazuru, Kokurakita-ku, Kitakyushu, Fukuoka 803-8580, Japan; ^3^Department of Cardiology and Periodontology, Kyushu Dental College, 2-6-1 Manazuru, Kokurakita-ku, Kitakyushu, Fukuoka 803-8580, Japan; ^4^Department of Clinical Communication Practice, Kyushu Dental College, 2-6-1 Manazuru, Kokurakita-ku, Kitakyushu, Fukuoka 803-8580, Japan

## Abstract

Bone defects often result from tumor resection, congenital malformation, trauma, fractures, surgery, or periodontitis in dentistry. Although dental implants serve as an effective treatment to recover mouth function from tooth defects, many patients do not have the adequate bone volume to build an implant. The gold standard for the reconstruction of large bone defects is the use of autogenous bone grafts. While autogenous bone graft is the most effective clinical method, surgical stress to the part of the bone being extracted and the quantity of extractable bone limit this method. Recently mesenchymal stem cell-based therapies have the potential to provide an effective treatment of osseous defects. In this paper, we discuss both the current therapy for bone regeneration and the perspectives in the field of stem cell-based regenerative medicine, addressing the sources of stem cells and growth factors used to induce bone regeneration effectively and reproducibly.

## 1. Introduction

Regenerative medicine is the medical field that creates functional tissues to repair and replace damaged or malfunctioning tissues and organs [1]. Tissues or organs generated from a patient's own cells would allow transplants without tissue rejection. Furthermore, regenerative medicine treatments have the potential to replace organ transplants or artificial organs. Because regenerative medicine generates tissues or organs using engineering technology, it is also called “tissue engineering.” To make regenerative medicine successful, three elements are required: stem cells, scaffolds, and growth factors [1]. Translational research, which takes results from the laboratory and translates them to the clinics, and industry-academic collaborations also play important roles in making regenerative medicine suitable for practical use.

**Figure 1:**
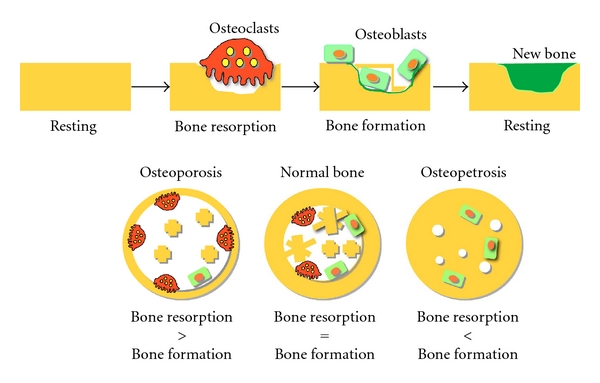
The schematic outlines of the bone remodeling cycle and the balance of bone resorption and bone formation. (a) In bone tissue, the osteoblasts are involved in new bone formation, while osteoclasts play a major role in bone resorption. The first step in the bone remodeling cycle is the resorption of existing bone by osteoclasts, followed by formation of the cement line in resorption lacunae and osteoblasts. Each cell type seems to be regulated by a variety of hormones and by local factors. (b) If the balance between bone formation and resorption is lost by the uncontrolled production of regulators, bone structure would be strikingly damaged, and the subject would be susceptible to osteoporosis and osteopetrosis.

The human skeleton consists of approximately 200 bones, and it weighs approximately 2 kg. Bone is a tough supporting tissue and functions in both movement and the maintenance of postural stability by working cooperatively with muscles. Bones also play an important role in calcium metabolism. Despite its hard structure, bone actually exists in a constant state of dynamic turnover known as bone remodeling [2] (Figure 1). There are two types of bone structures, cortical bone and cancellous bone. The ratio of the cortical bone and the cancellous bone of an adult is 9 : 1. Approximately 3% of the cortical bone is remodeled per year, whereas more than 30% of the cancellous bone is remodeled per year. Thus, approximately 6% of all human bones will be remodeled in a year. The bone mass in an adult human reaches its maximal level (peak bone mass) during a person's twenties and then gradually declines thereafter, as the speed of bone resorption exceeds bone formation with increasing age. Although bone mass in humans decreases by approximately 1% per year, the bone mass in women entering menopause will decrease by approximately 3% per year. At the remodeling sites, osteoblasts produce new bone, while osteoclasts resorb existing bone. Each cell type seems to be regulated by a variety of hormones and by local factors. If the balance between bone formation and resorption is lost by uncontrolled production of these regulators, the bone structure will be damaged, and the subject would be susceptible to osteoporosis and osteopetrosis [2] (Figure 1).

Bone defects often result from tumor resection, congenital malformation, trauma, fractures, surgery, or periodontitis in dentistry, as well as from diseases, such as osteoporosis or arthritis. The gold standard for reconstruction of large bone defects is the use of autogenous bone grafts [3]. This method has significant limitations, such as a lack of sufficient transplantable materials, donor site morbidity, inflammation, and resorption of the implanted bone. Although alternatives, such as the use of allografts or synthetic grafting materials, address these limitations, both alternatives are also limited by immunogenesis or lack of osteoinductivity [4].

The discovery of stem cells and recent advances in cellular and molecular biology have led to the development of novel therapeutic strategies that aim to regenerate tissues that were injured by disease. Recently, embryonic stem cells (ES cells) [5], induced pluripotent stem cells (iPS cells) [6], and somatic stem cells have been reported; however, there are many issues to overcome for the clinical use of ES and iPS cells, including ethical and safety problems, immunorejection, and tumorigenesis.

This review discusses the current therapies for bone regeneration and perspectives in the field of stem cell-based regenerative medicine, addressing sources of stem cells and growth factors to develop an efficient and high-quality bone derivation without any immunorejection, and tumorigenesis. We also discuss the potential use of regenerative medicine in dental tissue engineering.

## 2. Current Status of Bone Regeneration in Dentistry

Chronic dental disease and tooth loss often lead to the loss of hard tissue in the jaw. Patients with missing teeth by infections, or inflammation may experience bone resorption with loss of the affected part of the jaw. In addition to making a patient uncomfortable, this bone loss can cause unsightly disfigurements and may complicate the fitting of implants and other dental appliances. Although dental implants serve as an effective treatment to recover mouth function from tooth defects, many patients do not have adequate bone volume to build an implant. The outline of the current method to increase bone volume is described.

### 2.1. Bone Grafts and Artificial Bone Materials

Although the autogenous bone graft is the most effective clinical method for bone repair, it can be restricted by surgical stress to the site of bone extraction and the quantity of extractable bone. Demineralized and freeze-dried bone (DFDBa) extracted from the body is used for xenogamous bone grafts [3, 4].

Hydroxyapatite or various forms of *β*-tricalcium phosphate (*β*-TCP) are used as artificial bone materials [7]. Because these calcium phosphate materials do not have bone guidance capability, they are used together with autogenous bone grafts or other bone increasing methods, such as the guided bone regeneration (GBR) method and platelet-rich plasma (PRP).

### 2.2. Guided Bone Regeneration: GBR

Guided bone regeneration (GBR) encourages new bone growth to replace areas of damage in the jaw and can be used alongside guided tissue regeneration (GTR) to rebuild soft tissue in a patient's mouth [8]. The technologies and practices behind these techniques are subject to constant refinement, and clinical studies examine the possible application of these techniques to other regions of the body. GBR involves epithelial and connective tissue exclusion and space creation to allow the cells of the periodontal ligament to repopulate the root surface and to allow bone cells to grow into the area of the defect. GBR is usually performed together with a bone graft or PRP. Although this method induces self-regeneration of bone, it takes a long time to obtain adequate bone volume in many cases.

### 2.3. Distraction Osteogenesis

Distraction osteogenesis is a well-established technique used by orthopedic surgeons to repair long bone defects without the use of grafting materials and has gained acceptance over the past 15 years for the correction of various craniofacial deformities [9]. Several studies in various animal models demonstrated the application of osteodistraction at a number of different sites, including the mandible, the maxilla, the midface, and the cranial vault. There are several advantages of distraction osteogenesis over conventional osteotomy: operative times and blood loss are reduced, bone grafts are unnecessary, and bone is distracted in conjunction with the surrounding soft tissues and nerves. However, distraction osteogenesis has some disadvantages, such as technique-sensitive and equipment-sensitive surgery, and the possible need for a second surgery to remove distraction devices and patient compliance.

### 2.4. Platelet-Rich Plasma: PRP

In the field of dentistry, PRP has been used in different clinical procedures, such as sinus floor elevation, alveolar ridge augmentation, mandibular reconstruction, maxillary cleft repair, treatment of periodontal defects, and treatment of extraction sockets, where it has been applied alone or in addition to the autogenous bone, anorganic bone mineral, and organic bone substitutes [10]. The growth factors present in PRP are thought to contribute to the bone-healing process. The following growth factors are reported to be present in PRP: platelet-derived growth factor (PDGF), transforming growth factor-*β* (TGF-*β*), vascular endothelial growth factor (VEGF), epithelial growth factor (EGF), insulin growth factor-1 (IGF-1), and basic fibroblast growth factor (bFGF). In addition, three blood proteins, fibrin, fibronectin, and vitronectin, are known to act as cell adhesion molecules for osteoconduction [11, 12]. Therefore, PRP may influence bone formation through a variety of pathways.

## 3. A Cell-Based Therapy for Bone Regeneration in Dentistry

**Figure 2:**
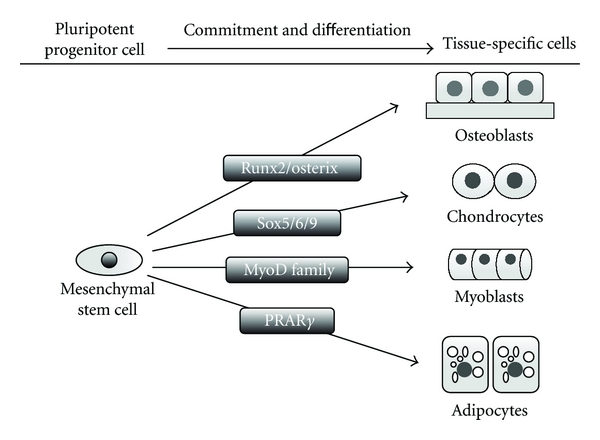
A schematic model for the differentiation of mesenchymal stem cells into tissue-specific cells by specific transcriptional factors. Mesenchymal stem cells can differentiate into osteoblasts, chondrocytes, myoblasts, and adipocytes. Each differentiation program is regulated by specific transcription factors: Runx2/Osterix, Sox5/6/9, MyoD family, and PPAR*γ*, respectively.

In recent years, stem cell research has grown exponentially due to the recognition that stem cell-based therapies have the potential to improve the life of patients with several kinds of diseases, such as Alzheimer's disease and cardiac ischemia. These therapies have also a role in regenerative medicine, such as the repair of bone or tooth loss. Stem cells have the potential to differentiate into several cell types, including odontoblasts, neural progenitors, osteoblasts, chondrocytes, and adipocytes (Figure 2). Mesenchymal stem cells (MSC) are multipotent progenitor cells that were originally isolated from various tissues, including adult bone marrow, adipose tissue, skin, umbilical cord, and placenta. Bone marrow-derived MSCs have been used in clinical trials for the effective treatment of osseous defects. However, bone marrow aspiration is an invasive and painful procedure for the donor and is a difficult procedure for a general practitioner. Furthermore, MSCs constitute heterogeneous cell types, and the potential for proliferation and differentiation of the MSCs depends on a patient's age, sex, or the presence of certain medical conditions, such as diabetes or hypertension [13].

Several cell populations with stem cell properties have been isolated from different parts of the tooth, including the pulp of both exfoliated and adult teeth, the periodontal ligament, and the dental follicle. Dental pulp stem cells (DPSCs) [14] and stem cells from human exfoliated deciduous teeth (SHED) [15] have generic mesenchymal stem cell-like properties, such as self-renewal and multilineage differentiation. DPSCs and SHED have the ability to generate not only dental tissue but also bone tissue. Because SHED exhibit higher proliferation rates and can be obtained with ease compared to bone marrow-derived MSCs, they might become an attractive source of autologous stem cells for bone regeneration. As described above, MSCs are heterogeneous cell populations; therefore, to induce bone regeneration effectively and reproducibly, it is important to understand the mechanisms by which growth factors or cytokines regulate osteoblast differentiation.

### 3.1. Regulation of Osteoblast Differentiation

Bone consists of hydroxyapatite crystals and various kinds of extracellular matrix proteins, including type I collagen, osteocalcin, osteopontin, bone sialoprotein and proteoglycans. Most of these bone matrix proteins are secreted and deposited by mature osteoblasts, which are aligned on the bone surface [2, 16]. The formation of hydroxyapatite crystals in osteoids is also regulated by osteoblasts. Therefore, the expression of a number of bone-related extracellular matrix proteins, the high enzyme activity of alkaline phosphatase (ALP) by responses to osteotropic hormones and cytokines are believed to be major characteristics of osteoblasts [2, 16].

It is well known that osteoblasts, chondrocytes, adipocytes, myoblasts, tendon cells, and fibroblasts are differentiated from common precursors in the bone marrow-derived MSCs. The lineages are determined by different transcription factors. The transcription factors Runx2, Osterix, or *β*-catenin regulate osteoblast differentiation, the Sox family of transcription factors (Sox9, Sox5, and Sox6) regulate chondrocyte differentiation, MyoD transcription factors (MyoD, Myf5 and Myogenin) regulate myogenic differentiation, and the C/EBP family (C/EBP*β*, C/EBP*δ*, and C/EBP*α*) and PPAR*γ* transcription factors regulate adipocyte differentiation (Figure 2). Runx2 directs multipotent mesenchymal cells to an osteoblastic lineage, and *β*-catenin, Osterix, and Runx2 direct them to mature osteoblasts after differentiation to preosteoblasts [2, 16, 17].

Several hormones and cytokines, such as bone morphogenetic proteins (BMP), TGF-*β*, Wnt, hedgehog, bFGF, and estrogen, are involved in the regulation of mesenchymal cell differentiation by stimulating intracellular signaling pathways. Among them, BMP is one of the most powerful cytokines to induce ectopic bone formation, and it strongly promotes the differentiation of mesenchymal cells into osteoblasts.

BMPs, members of the TGF-*β* superfamily, were originally identified by their ability to induce ectopic bone formation when implanted into muscle tissue, and they play a pivotal role in the signaling networks and processes associated with skeletal morphogenesis [16, 17]. BMP signals are transduced from the plasma membrane receptors to the nucleus through both the Smad pathway and non-Smad pathways and are regulated by many extracellular and intracellular molecules that interact with BMPs or components of the BMP signaling pathways. This bone-inducing ability of BMPs should be useful for the development of bone regeneration. However, BMPs cannot generate a sufficient clinical response to be used in bone regeneration. One possible reason might be that inflammatory cytokines inhibit both bone formation and osteoblast differentiation induced by BMPs. For example, the inflammatory cytokine tumor necrosis factor (TNF) *α* inhibits osteoblast differentiation in multiple models, including fetal calvaria, bone marrow stromal cells, and osteoblastic cells [18–20].

### 3.2. Inflammatory Cytokines Suppress Osteoblast Differentiation

Inflammatory cytokines, such as TNF*α* or interleukin-(IL-)1, contribute to local and systemic bone loss in inflammatory bone diseases, such as rheumatoid arthritis and periodontitis, and estrogen deficiency [21]. In patients with rheumatoid arthritis, TNF*α* and other cytokines are overproduced in inflamed joints by various cells that infiltrate the synovial membrane, and anti-TNF drugs, such as infliximab, etanercept, and adalimumab, have been shown to not only diminish signs and symptoms of disease but also to prevent joint damage [22]. Under these conditions, osteoblast-mediated bone formation cannot compensate for accelerated osteoclastic bone resorption, suggesting a direct inhibitory effect of inflammatory cytokines on osteoblasts.

Consistent with clinical and *in vivo* animal studies, the inhibitory effects of TNF*α* or IL-1*β* on bone formation *in vitro* were also observed with a neonatal rat calvarial organ culture system [16]. TNF*α* or IL-1*β* inhibited not only spontaneous osteoblast differentiation but also BMP-induced osteoblast differentiation, as measured by a change in the BMP2-induced expression of Runx and osteocalcin and a dose-dependent change in ALP activity. These responses were mediated *via* several signaling pathways, such as mitogen-activated protein kinase (MAPK), extracellular signal regulated kinase (ERK), c-Jun N-terminal kinase (JNK), p38 kinase, and NF-*κ*B.

### 3.3. Suppression of NF-*κ*B Enhances BMP-2-Induced Osteoblast Differentiation

The transcription factor NF-*κ*B has a key role in inflammation and immune responses. Previous studies have shown that inhibition of NF-*κ*B suppresses inflammatory bone loss by inhibiting osteoclastogenesis in an arthritis model, suggesting that NF-*κ*B is a major target of inflammatory bone diseases [23]. The importance of NF-*κ*B in osteoblasts was revealed in a recent paper, where the authors expressed a dominant negative form of IKK*β* to inhibit NF-*κ*B in the mature osteoblasts of mice. Expression of this dominant negative IKK*β* led to increased BMD and bone volume due to the increased activity of osteoblasts [24].

**Figure 3:**
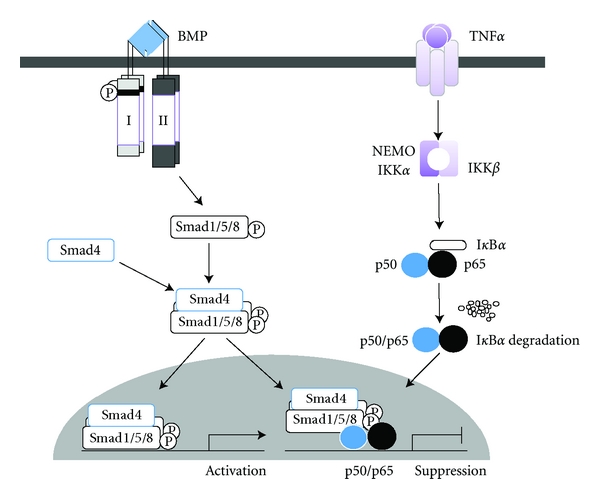
A model of NF-*κ*B-mediated inhibition of BMP/Smad-mediated DNA binding activity. NF-*κ*B, particularly the p65 subunit, binds the Smad1/Smad4 complex directly or indirectly, and that this binding interferes with the DNA binding of Smad proteins induced by BMP-2.

**Figure 4:**
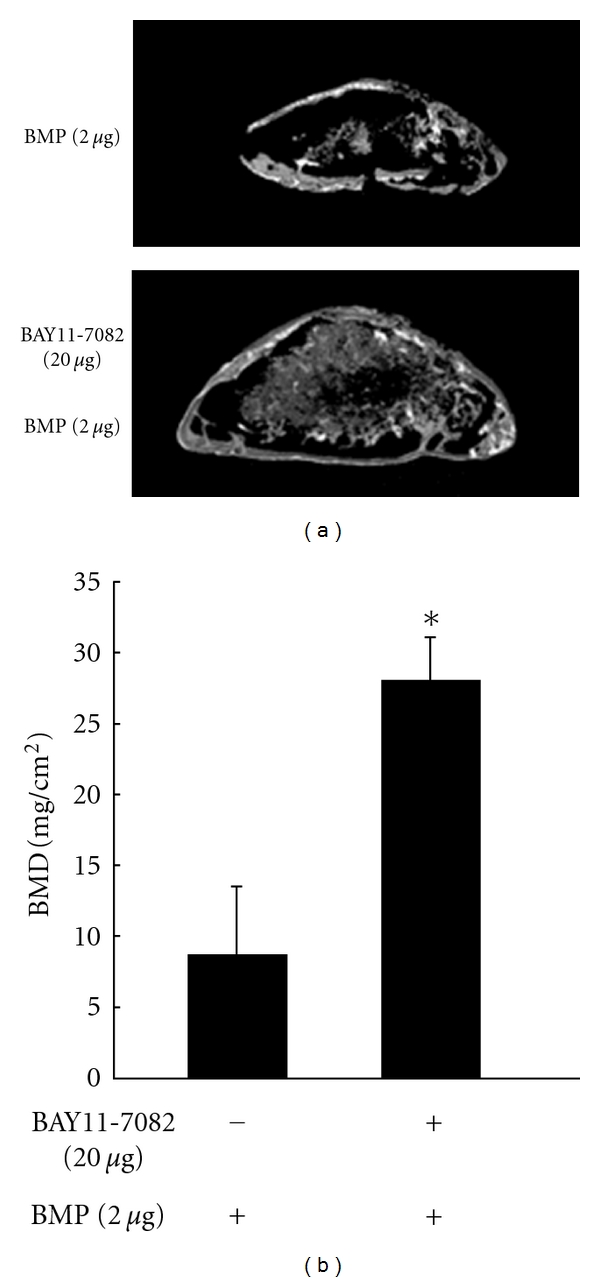
BMP2-induced ectopic bone formation *in vivo* is enhanced in the presence of a selective inhibitor of NF-*κ*B, BAY11-7082. Two micrograms of BMP2 was implanted subcutaneously to induce ectopic bone formation in the presence or absence of BAY11-7082 in mice (*n* = 8). (a) *μ*CT reconstruction images of ectopic bone in the presence or absence of BAY11-7082 in mice. Bar: 1 mm. (b) Bone mineral density (BMD) of the ectopic bone in the presence or absence of BAY11-7082 was measured by dual-energy X-ray absorptiometry (DXA). *P < 0.01.

Inhibition of NF-*κ*B by overexpression of the dominant negative form of I*κ*B*α* (I*κ*B*α*DN) leads to the induction of osteoblast differentiation [25]. The cell permeable NF-*κ*B activation antagonist TAT-NBD blocks the activation of NF-*κ*B by TNF*α* and could prevent TNF*α* from suppressing TGF*β*-stimulated Smad luciferase activity, BMP2-induced Runx2 mRNA expression, and osteoblast differentiation in MC3T3-E1 cells, a mouse osteoblastic cell line [26]. Furthermore, the selective inhibition of NF-*κ*B increased the bone formation and ameliorated osteopenia in ovariectomized mice [27]. We have previously shown that TNF*α* inhibited BMP-induced osteoblast differentiation through NF-*κ*B activation by inhibiting Smad DNA binding [28] (Figure 3). Therefore, we examined whether the selective inhibitor of NF-*κ*B, BAY11-7082, enhanced the ectopic bone formation induced by BMP2 in mice. BMP2-induced ectopic bones were enlarged and had enhanced radioplaques in the presence of BAY11-7082 compared with BMP2 treatment alone. The *μ*CT image of ectopic bones induced by BMP2 together with BAY11-7082 showed a thick outer bone filled with trabecular bone (Figure 4(a)). The bone mineral density (BMD) of these ectopic bones were also increased in the presence of BAY11-7082 (Figure 4(b)). These results strongly indicate that inhibition of NF-*κ*B may promote BMP-induced bone regeneration in the treatment of bone diseases.

## 4. Conclusion

Although regenerative medicine has been tried in various fields, there is much demand for regenerative medicine in dentistry, particularly in bone regeneration. Depending on the state of periodontitis or jaw resection, it might take more than 6 to 12 months for occlusal reconstitution. Thus, the development of an efficient and high-quality bone derivation method is necessary. Cell-based therapy may pave the way to rejection-free regenerative treatment for bone defects. It is also likely that research concerning growth factor or cell-based therapies will continue to progress. However, there are also many problems, such as laws and costs of equipment, that must be solved. Although it is unclear when the technology of regenerative medicine will be put into practical use, it is important to follow the current status of regenerative medicine to keep abreast of the progression the technology.
